# Supplementary material for: A Critical View of the Application of the APEX Software (Aqueous Photochemistry of Environmentally-Occurring Xenobiotics) to Predict Photoreaction Kinetics in Surface Freshwaters
Source: Molecules. 2019 Dec 18;25(1):9. doi: 10.3390/molecules25010009 (PMC7017383; doi:10.3390/molecules25010009)
Supplement: Supplementary file 1 [file molecules-25-00009-s001.zip › 3.2.4_gcc-4.4.0.zip!/3.2.4_gcc-4.4.0/3.2.4_gcc-4.4.0/doc/octave/html/interpreter/Errors-and-Warnings.html]

Errors and Warnings - Untitled


Next: Debugging,
Previous: Functions and Scripts,
Up: Top


---

## 12 Errors and Warnings

Octave includes several functions for printing error and warning
messages. When you write functions that need to take special action
when they encounter abnormal conditions, you should print the error
messages using the functions described in this chapter.

Since many of Octave's functions use these functions, it is also useful
to understand them, so that errors and warnings can be handled.

- Handling Errors- Handling Warnings
